# Cancer Treatment Patterns Among Yukon Residents Referred to British Columbia for Care: A 13-Year Retrospective Study

**DOI:** 10.3390/curroncol32110641

**Published:** 2025-11-16

**Authors:** Kaylie Willemsma, Jonathan Simkin, Debon Lee, Emma Quinn, Kira Makuk, Emily B. Jackson, Andrew Bang, Manik Chahal, Ying Wang, Jessica Chan

**Affiliations:** 1 Department of Medical Oncology, BC Cancer, 600 W 10th Avenue, Vancouver, BC V5Z 4E6, Canada; 2 BC Cancer Registry, BC Cancer, 600 W 10th Avenue, Vancouver, BC V5Z 4E6, Canada; 3 Population and Public Health, Evidence and Evaluation, Health and Social Services, Government of Yukon, 1191 Front Street, Whitehorse, YT Y1A 0K5, Canada; 4 Department of Radiation Oncology, BC Cancer, 600 W 10th Avenue, Vancouver, BC V5Z 4E6, Canada; 5 Department of Surgery, University of British Columbia, 2329 West Mall, Vancouver, BC V6T 1Z4, Canada

**Keywords:** Yukon, breast, colorectal, prostate, lung, rural medicine, timepoint, healthcare access

## Abstract

Yukon residents often must travel long distances to access specialized cancer care, which may impact cancer treatment patterns. We conducted a retrospective study to characterize all adult breast, prostate, colorectal, and lung cancer cases from the Yukon, diagnosed from 2009 to 2021 and seen in consultation at BC Cancer. We collected data on demographics, tumour characteristics and treatment, including timepoints for cancer care. A secondary analysis of non-referred cases was conducted. There were a total of 336 breast, 270 prostate, 279 colorectal and 266 lung cancer cases diagnosed in the Yukon from 2009 to 2021, of which 298 (88.7%), 120 (44.4%), 206 (73.8%) and 204 (76.7%) cases were referred to BC Cancer, and 266 (79.2%), 118 (43.7%), 204 (73.1%) and 183 (68.8%) were included in this study, respectively. Most cases were diagnosed at an early stage (breast: 92.9%, prostate: 82.2%, colorectal: 72.1%, lung: 45.9%). Nearly 70% of cases resided in Whitehorse (Yukon’s capital), where most Yukon residents live. Compared to available published Canadian timepoints, Yukon patients had similar or shorter wait times in 13 of 22 timepoints along the pathway to diagnosis and treatment. However, time from biopsy to surgery had the longest relative wait times across all tumour groups (range: 26–60% longer). Our study provides baseline data that can help inform cancer care provision for Yukon residents.

## Simple Summary

Residents of the Yukon who are diagnosed with cancer often must travel out of territory to receive cancer care, and many are seen and treated at BC Cancer in British Columbia. The purpose of our study was to describe the cancer and treatment characteristics of Yukon residents diagnosed with either breast, prostate, colon or lung cancer from 2009 to 2021. We found that approximately two-thirds of people in the Yukon diagnosed with cancer received cancer care through BC Cancer. Most cancer cases were diagnosed at an early stage and most patients lived in the capital of Whitehorse. Yukon residents in this study experienced shorter wait times in certain parts of the diagnosis and treatment pathway compared to other Canadian studies; however, some of the longest wait times were seen across all tumour groups from date of biopsy to date of surgery. Results from this study can help inform approaches to strengthen cancer service delivery in the Yukon.

## 1. Introduction

**Figure 1:**
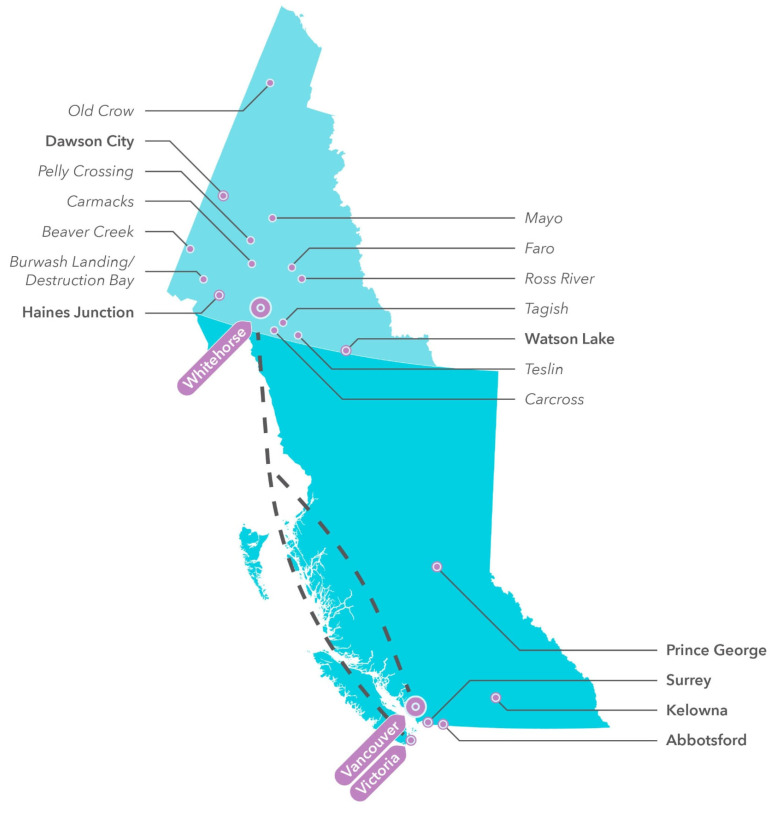
Map of Yukon communities of >100 people in 2021 and BC Cancer referral sites [5]. Dotted lines represent available direct flights from Whitehorse.

The Yukon is the northwestern-most territory in Canada, with a population of 33,733 in 2009, growing to 42,961 in 2021 [1]. Cancer is the leading cause of death in the Yukon, with approximately 70 cancer deaths annually [2]. Residents of the Yukon who are diagnosed with cancer are often required to travel out of territory to see oncology specialists and receive treatment. BC Cancer is the provincial cancer care provider for British Columbia (BC; a province located immediately south of the Yukon), with a mandate that includes cancer care for both BC and Yukon residents; thus, the majority of Yukon cancer patients are referred to one of BC Cancer’s six regional centres [3,4]. This requires a flight time of just over 2 h from the capital city of Whitehorse to a referral centre either in Victoria or Vancouver, BC (Figure 1). A limited number of patients may choose to travel to other provinces for cancer care. Another subset of patients may receive systemic treatment and be followed by General Practitioners in Oncology (GPOs) at Whitehorse General Hospital in the Yukon, in a collaborative care model with a treating oncologist at BC Cancer [4].

The Yukon’s capital of Whitehorse is the only urban centre in the territory, and represents 70% of the territory’s population [6]. The previous literature has described the geography and weather, which lead to limited and inconsistent access to cancer care for individuals living in Canada’s territories. This includes needing both time and financial resources to travel south for diagnostic tests (i.e., PET scans, MRIs, certain biopsies), for all oncology consultations, all radiotherapy treatments and certain systemic therapies [7,8,9,10,11]. While the government provides financial support systems for Yukon patients and an escort, to partially reimburse flights, meals and accommodation, these programmes require an application and healthcare provider approval [9,11,12,13,14]. Indigenous patients receive financial support through the Non-Insured Health Benefits (NIHB) programme, which differs in eligible expenses and amount reimbursed [9,13,14].

In addition, Indigenous peoples represent 22% of the Yukon’s total population in 2021 [15]. Indigenous peoples globally experience colonial, cultural and systemic barriers to cancer care, which include harmful interactions with the healthcare system leading to mistrust, language barriers, traditional practices that are not accepted in western medicine and increased travel duration and cost to access healthcare [2,8,9,16,17,18]. These challenges can lead to delayed cancer diagnosis, advanced stage at diagnosis or poorer outcomes [8,10,19]. Ultimately, this can affect quality of life, with patients having also reported difficulty being away from family/community supports during cancer care [8,11,20].

Specific to the Yukon, however, there are very limited studies looking at patterns of cancer diagnosis and treatment. A 2019 report by the Government of Yukon showed that between 2009 and 2016, there was an average of 153 new cancer cases diagnosed in the Yukon annually [2]. Breast, prostate, colorectal and lung cancer were the four most common cancers in the Yukon, which mirrored national patterns [2,21]. Cancer incidence was either similar or less than the Canadian average, by cancer type, with the exception of female breast cancers, which were diagnosed at higher rates in the Yukon [2]. Overall, after correcting for population ageing and growth, the incidence of cancer was found to be decreasing in the Yukon [2]. Two studies have reported on Yukon cancer mortality and found poorer survival outcomes in the four most common cancers compared to the rest of Canada [2,22]. It has been postulated that lack of access to timely cancer diagnostics and care may contribute to the poorer survival outcomes seen amongst this population [2,22]. However, cancer care treatment patterns for residents of the Yukon have not yet been described.

Therefore, the aim of this study is to characterize the current demographic, tumour factors and treatment patterns (including timeline of treatment) of Yukon residents referred to BC Cancer. A secondary analysis was performed to describe the age, location, cancer type and stage of cases not referred to BC Cancer. This study is a first step to understanding access to cancer care for this population and can ultimately be used to provide the groundwork for future studies which can inform cancer service delivery and planning.

## 2. Materials and Methods

This was a retrospective descriptive study of all adult (aged ≥ 18 years) Yukon residents with a diagnosis of invasive cancer of the breast (infiltrating ductal carcinoma, infiltrating lobular carcinoma), prostate (prostatic adenocarcinoma), colorectal (colorectal adenocarcinomas) or lung cancer (small cell carcinoma, squamous and non-squamous non-small cell lung cancer subtypes) between January 2009 and December 2021 and seen in consultation at BC Cancer. BC Cancer has six sites across the province: Vancouver, Victoria, Kelowna, Surrey, Prince George and Abbotsford. Yukon patients were referred to BC Cancer through the standard referral form—the same form is used for BC and Yukon residents [23]. Reasons for exclusion were not being referred to BC Cancer, pathology showing in situ disease, pre-malignancy or non-malignancy or lack of tissue confirmation of malignancy. Similarly, exclusion criteria for the non-referred cases were pathology showing in situ disease, pre-malignancy or non-malignancy or lack of tissue confirmation of malignancy.

This study investigated cancer cases rather than patients, as some patients had multiple cancer diagnoses which were collected and analyzed individually. Cases were identified through the Yukon Cancer Registry. A manual chart review of all cancer cases using patient health numbers was conducted through BC Cancer’s electronic medical record systems to supplement data from the Yukon Cancer registry. We collected data on demographics (age, sex, postal code, BC Cancer referral site), tumour characteristics (stage, grade, histologic subtype), and treatment (surgery, systemic therapy, radiotherapy). Cancer staging data was categorized as early-stage (stage I-III) versus late-stage (stage IV). Systemic therapy data included use of chemotherapy, targeted therapy, hormone therapy and immunotherapy, as well as the intent (curative vs. palliative) and number of lines of treatments. Radiotherapy data included treatment intent (curative vs. palliative) and site of treatment (regional vs. distal). Chemotherapy and surgery data included treatments that occurred both in BC and Yukon, while radiotherapy was exclusively administered in BC in this study population. Timepoint data included date of first investigation (imaging or exam suspicious for cancer), diagnostic biopsy, oncologist consultation, surgery, first chemotherapy and first radiotherapy. The term “first intervention” was used to capture the earliest date of either date of surgery, chemotherapy start date or radiotherapy start date. The term “earliest consultation” was used to capture the earliest date a patient was seen by either a medical or radiation oncologist in consultation at BC Cancer. Timepoints for early, late and all stages were compared to Canadian published benchmarks and retrospective studies. A timepoint from our study was considered similar to the published literature if one of three criteria were met: the median study timepoint (either early-, late- or all-stage) was identical to the published timepoint, the median study timepoint (either early-, late- or all-stage) fell within a range of published timepoints, or if the study median timepoints by stage were inconsistently below or above the published timepoint.

Descriptive analyses were performed separately for breast, prostate, colorectal and lung cancer cohorts, including means, median, ranges and proportions for the variables listed above. A secondary analysis was conducted to descriptively compare characteristics (sex, geography, age and stage) among Yukon cancer cases that were referred to BC Cancer versus not referred. Histology data among non-referred cases were not consistently available and an analysis could not be completed. Descriptive statistics for variables with fewer than five values were suppressed to protect patient privacy.

This study was approved by the University of British Columbia—BC Cancer Research Ethics Board (H23-01629).

## 3. Results

**Figure 2:**
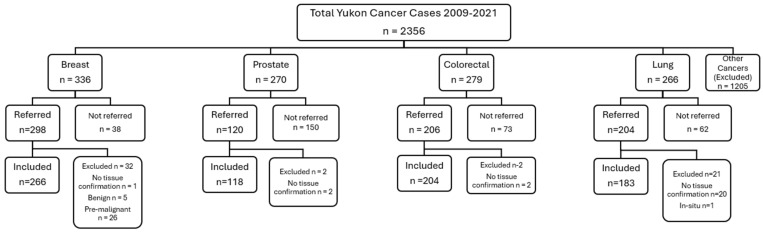
Consort diagram of cases included in the study.

A total of 2356 cases across all cancer types were diagnosed in the Yukon between 2009 and 2021, of which 1520 cases (64.5%) were referred to BC Cancer. There were a total of 336 breast, 270 prostate, 279 colorectal, and 266 lung cancer cases diagnosed, of which 298 (88.7%), 120 (44.4%), 206 (73.8%) and 204 (76.7%) cases were referred to BC Cancer, respectively. Of the total diagnosed cases, 771 cases met inclusion criteria and were included in this study: 266 (79.2%) breast, 118 (43.7%) prostate, 204 (73.1%) colorectal and 183 (68.8%) lung cancer cases (Figure 2).

There were 41 patients with more than one case of either breast, prostate, colorectal or lung cancer during this study period. Of the cases included in this study, 22 (8.3%) breast cases, 1 (0.8%) prostate case, 5 (2.5%) colorectal cases and 10 (5.5%) lung cases occurred in patients with more than one case of cancer.

### 3.1. Study Cohort Characteristics

The median age of this study population was 59, 67, 64 and 68 years for breast, prostate, colorectal and lung cancer cases, respectively (Table 1). There were no male cases of breast cancer, while 42% of colorectal and 46% of lung cancer cases were among males. Over 88% of cases were referred to BC Cancer, Vancouver, and approximately two-thirds of cases resided in the Yukon’s capital of Whitehorse. Among breast cancer cases, 74.3% were estrogen and progesterone receptor-positive and HER2-negative. Mutation status of targetable genes was unknown in approximately 50–75% of colorectal and lung cancer cases. Among colorectal cases, 9.8% were KRAS-positive, 2.9% were BRAF-positive and 11.3% were MMR-positive. Among lung cancer cases, <5% were EGFR positive, 15.1% were KRAS positive, and 22.3% of NSCLC cases had a PDL1 of >1%, of which 14.2% were >50%. There were no identified cases of ALK or ROS1 translocation amongst lung cancer cases. Median initial PSA was 7.6 (range 1.2–1400) among the 115 prostate cancer cases with documented PSAs (Supplemental Table S1).

The majority of cases were early-stage for breast, prostate and colorectal cases (92.9%, 82.2% and 72.1%, respectively), while 45.9% of lung cancer cases presented at an early stage. Regarding grade, 30.5% of breast cancer cases and 43.2% of prostate cancer cases presented with high-grade disease.

Among breast cancer cases, 253 (95.1%) had surgery, 204 of the 223 (91.5%) hormone-positive breast cancer cases received hormone therapy, 125 (47.0%) had chemotherapy (of which 75% were with curative intent), 37 (13.9%) had targeted therapy and 153 (57.5%) had radiotherapy (of which 87% were with curative intent).

Among prostate cancer cases, 41 (34.7%) had surgery, 74 (62.7%) had androgen-deprivation therapy, <5 had chemotherapy (all of which were with palliative intent) and 87 (73.7%) had radiotherapy (of which 71% were with curative intent). There were 114 courses of external beam radiotherapy and 23 courses of brachytherapy among prostate cancer cases.

Among colorectal cancer cases, 149 (73.0%) had surgery (of which 40.3% had a hemicolectomy), 127 (62.3%) had chemotherapy (of which 59% were with curative intent), 43 (21.1%) had targeted therapy, <5 (<3%) had immunotherapy and 61 (29.9%) had radiotherapy (of which 70% were with curative intent).

Among lung cancer cases, 27 (14.8%) had surgery, 80 (43.7%) had chemotherapy (of which 33% were with curative intent), 18 (9.8%) had immunotherapy and 125 (68.3%) had radiotherapy (of which 20% were with curative intent).

### 3.2. Non-Referred Cases

Similarly to the referred cases, the majority of cases not referred to BC Cancer were diagnosed at an early stage: 80.0% of breast, 56.7% of prostate, 70.2% of colorectal and 52.1% of lung non-referred cancer cases (Table 2). There was, however, a lower percentage of early-stage prostate cancer diagnoses in the non-referred cases compared to the study cohort (57% vs. 82%). Non-referred cases were primarily male (223, 69.0%), of which 150 of these cases were prostate cancer cases. Of the non-referred colorectal cases, 60.3% were among males and of the non-referred lung cancer cases, 46.8% were among males. Non-referred cases had a higher median age compared to referred cases, except for prostate cancer cases, where median age was similar. The proportion of cases residing in Whitehorse compared to outside of Whitehorse was similar in referred and non-referred cases. Overall, the proportion of cases referred to BC Cancer was stable over the course of the study period.

### 3.3. Timepoints of Cancer Care

Overall, cases with early-stage disease experienced longer wait times than those with late-stage disease, with the exception of three timepoints: biopsy to first intervention for colorectal cancer, referral to radiation oncology consultation for colorectal cancer and medical oncology consultation to start of chemotherapy for lung cancer, where late-stage cases waited longer (Supplemental Table S2a–e). The shortest median time between the first investigation of cancer and diagnostic biopsy was observed in breast cancer cases, with 23 days for early-stage and 22 days for late-stage. In comparison, for prostate cancer cases, the median times were 93 days for early-stage and 63 days for late-stage, and 45 days for early-stage and 25 days for late-stage for lung cancer cases. There was no data available on first investigations for the colorectal cases. Lung cancer cases had the shortest median time to an oncology consultation following diagnostic biopsy with 23 days for early-stage and 19 days for late-stage, while there was a median time of 83 days and 47 days for early- and late-stage breast, 64 days and 52 days for early- and late-stage prostate, and 55 days and 35 days for early- and late-stage colorectal cancer cases. Breast and colorectal cases had shorter timepoints from biopsy to surgery than biopsy to consultation, as many of these cases had surgery prior to being seen by an oncologist. Wait times from biopsy to first intervention varied across the four tumour groups. For breast cancer cases, the median timepoint was 42 days in early-stage cases versus 32 days in late-stage. For prostate cancer cases, timepoints were more prolonged for early-stage (98 days) compared to late-stage (29 days). Colorectal cancer cases showed the opposite pattern, with a median of 38 days for early-stage and 47 days for late-stage. For lung cancer cases, early-stage cases had a median timepoint of 48 days, while late-stage cases was 29 days.

## 4. Discussion

This study provides a comprehensive overview of the referred cases of the four most common cancers diagnosed in the Yukon over a thirteen-year period. The demographic data of this study mirrors a previously published report in 2016 from the Government of Yukon regarding cancer incidence in the territory [2].

### 4.1. Referral Patterns

The referral rates in this study from Yukon to BC Cancer for all cancer types were similar to referral rates to BC Cancer published in 2017 (64.5% vs. 64.6%), which included both BC and Yukon residents in their cohort [24]. There were interesting differences in the rates in this study compared to BC Cancer’s published rates by cancer type; while rates were similar for breast cancer (88.7% vs. 89.8%) and higher for lung (76.7% vs. 68.5%) and colorectal cancer (73.8% vs. 66.8%), they were lower for prostate cancer (44.4% vs. 51.5%) [24]. This study’s referral rates were also similar to rates published for out-of-territory oncology services in other territories, such as from Nunavut to Ontario (70.0%) [25].

A smaller proportion of cases were diagnosed at a late stage in this study compared to the Yukon-specific literature: 23.0% of breast, 50.6% of prostate, 42.5% of colorectal and 69.3% of lung were diagnosed at a late stage in the 2019 Yukon Cancer Incidence Report, compared to 7.1%, 17.8%, 27.9% and 54.1% of cases in our study, respectively [2]. This could be partially explained by differing definitions used to define early-stage versus late-stage. In this study, late-stage was defined as stage IV only, whereas in the Yukon Cancer Incidence Report defined late-stage as stages III and IV [2].

While breast cancer screening has long been available in the Yukon, colorectal screening was recently started in 2016 [26,27]. The data show an increase in the annual number of colorectal cases referred to BC Cancer after 2016 compared to prior. There is scarce data on the uptake of colon cancer screening in the Yukon; however, past Canadian cancer screening data suggests no significant difference in screening rates by geographic location within Canada [10,27,28]. This study also found a slight predominance in referred male colorectal and lung cancers compared to female. Interestingly, the portion of male vs. female cases were similar in non-referred and referred colorectal cases, though the previous literature from BC suggested that female colorectal cases were less likely to be referred [29]. Meanwhile, female Yukon residents with lung cancer were less likely to be referred to BC Cancer compared to their male peers in this study (53.2% non-referred cases were female vs. 45.9% of included cases were female).

Reasons for non-referral were not available in this study; however, they are likely multifactorial. Patient preference may play a role, given that being treated at BC Cancer requires travel out of territory, separation from one’s support system and treatments that may have significant morbidity [8,9,10,11,12,20,30,31]. While reimbursement programmes exist through the government, they are not eligible to cover the entire cost of travel [9,11,12,13,14]. Given that approximately half of the Indigenous population in the Yukon live outside of Whitehorse, longer travel journeys are often required. As such, factors such as cost, travel logistics and ability to bring an escort are of increased importance when considering cancer management options [9,14,32]. Physicians may also be less likely to refer if the patient has poor functional status or advanced age [12,29,33,34]. The median age was higher amongst non-referred cases in this study compared to cases included (64–71 vs. 59–68 years old). Finally, breast cancer and prostate cancer may be managed with palliative intent in the Yukon with hormone therapy, without need for BC Cancer referral [4].

Interestingly, prostate cancer had lower rates of referral compared to the other cancer types in this study (44.4% vs. >72%). Published rates of referral to BC Cancer of all diagnosed prostate cancer cases in BC and the Yukon was 51.5% in 2017, suggesting higher rates of referral of prostate cancer from BC than from the Yukon [24]. Non-referred cases had a higher proportion of diagnosis at a late stage compared to referred prostate cancer cases (43.3% vs. 17.8%). One explanation could be that patients with late-stage disease are less likely to travel and could be managed locally with systemic treatment such as ADT [4,35]. Non-referred prostate cancer cases had the same median age and similar mean age compared to referred prostate cancer cases, which makes age a less likely explanation for the marked decrease in referral rates in prostate cancer cases. There is mixed Canadian and BC data regarding radiotherapy referrals for prostate cancer cases, with some studies showing an increased rate and others showing decreased rates of referrals for rural patients compared to their urban counterparts [31,36,37,38,39,40,41,42]. Two studies found that as more rural radiation centres opened, there was higher rates of radiotherapy among rural patients, suggesting travel distance was contributing to low referral rates [35,41]. The Surveillance, Epidemiology, and End Results (SEER) Program in the United States has published numerous studies over time that have consistently shown that rural prostate cancer patients were more often untreated or observed compared to urban patients, but did not postulate reasons for this finding [43,44,45].

### 4.2. Geography

This study found that approximately 70% of referrals to BC Cancer from the Yukon originated from Whitehorse, which is aligned with the fact that 70% of Yukoners live in Whitehorse [6]. Similarly, 67% of non-referred cases resided in Whitehorse. The similarity in urban versus rural referral rates between non-referred and referred cases is encouraging; however, further work is needed to understand whether patient experiences and treatment utilization differ based on geography. Notably, there are two direct flights from the Yukon to BC: from Whitehorse to either Victoria or Vancouver. Consequently, the travel duration for those living outside of Whitehorse would be even greater.

Studies performed among neighbouring territories have sought to describe the cultural and geographic factors affecting access to cancer care, such as availability of flights, needing to relocate away from community for care and reduced accessibility with inclement weather [2,7,8,10,11]. There may also be language barriers and differences in cultural health beliefs among people from the Yukon seeking care in BC [9,11,46]. Studies have shown that individuals living in rural locations often face barriers that contribute to poorer health outcomes and disparities in cancer care, such as limited access to screening facilities close to home and decreased availability and therefore use of radiotherapy and palliative care [40,47,48]. In the Yukon, many health services are available in the urban centre of Whitehorse; however, there are no radiotherapy services [4].

Though the association of geography and cancer outcomes has not been studied in this population, we know from previous studies examining all-cancer mortality that Yukon males with cancer have statistically significantly poorer survival outcomes compared to the rest of Canada [2]. Age-standardized mortality rates in the Yukon were also significantly elevated in patients with prostate, breast, lung (females only), colorectal and gastric cancers compared to their BC counterparts [22]. In this same study, cancer survival outcomes for Yukoners were similar to those of BC residents residing in Northern BC, though a clear urban–rural trend could not be established as other rural regions of BC did not see the same elevated mortality rates [22]. Mortality data was not available for our current study and requires further investigation.

Virtual medicine has become increasingly common since the COVID 19 pandemic in March 2020 [49,50,51,52]. The substitution of in-person with telemedicine BC Cancer appointments, and the expansion of the collaboration between BC Cancer and GPOs in Whitehorse, has resulted in decreased travel time and cost for cancer patients, especially those from rural settings [4,49,50,51,52]. As a result, Yukon residents and rural BC residents surveyed have favoured the shift toward telemedicine [49,50,51,52,53].

### 4.3. Timepoints

**Figure 3:**
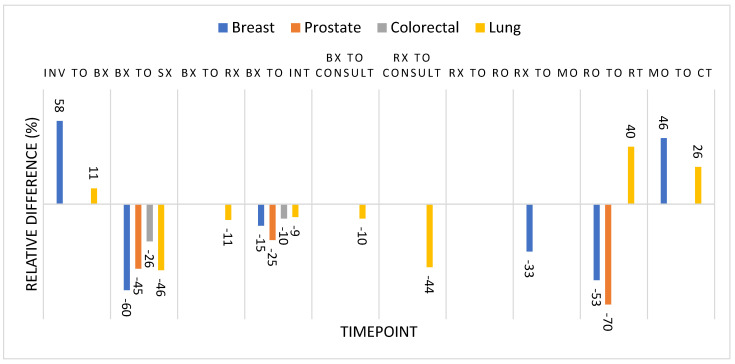
Relative difference between median timepoints in published studies compared to this study by cancer type. Negative relative differences represent longer wait times in this study compared to the literature. Inv = first investigation, Sx = surgery, Rx = referral, Int = first intervention, Consult = earliest consultation RO = radiation oncology, MO = medical oncology, RT = radiotherapy, CT = chemotherapy, Bx = biopsy, Rx = referral.

For the purposes of this discussion, diagnosis date will be equivalent to biopsy date. As seen in Supplemental Table S2a–e, there are limited and inconsistent timepoint benchmarks for cancer treatment in Canada [54,55,56,57,58,59,60,61,62,63,64,65,66,67]. There were comparison data for 19 tumour-specific timepoints and 3 all-cancer timepoints. The cases in this study had longer times to treatment in 9 of 22 timepoints, shorter times to treatment in 2 of 22 timepoints, and similar times in 11 of 22 timepoints. Overall, early-stage cancer cases had longer timepoints compared to their late-stage counterparts, which is in keeping with the published literature [55]. The longest relative wait times were seen from biopsy to surgery across all tumour groups, ranging from 26% to 60% longer in this study compared to the median target times found in the literature [62,63,64,67] (Figure 3). Relatedly, biopsy to first intervention was also longer across all tumour groups [60,64,65,67]. Wait times were also relatively longer from radiation oncology consult to start of radiotherapy for prostate and breast cancer cases [55,56,57,58]. However, patients with prostate cancer often start treatment with hormone therapy prior to radiotherapy, which can artificially increase this timepoint. Patients with early-stage breast cancer treated in BC follow a specific provincial protocol which allows for a longer targeted time to receiving radiotherapy without adversely impacting outcomes [56]. Patients from the Yukon would also be treated following the same protocol, and thus the time to receiving radiotherapy would be expected to be higher compared to the published literature. Encouragingly, time from investigation to biopsy and time from medical oncology consult to start of chemotherapy were shorter for the Yukon cancer cases in this study compared to published benchmarks [57,58,59,60,61,64,65,66,67].

Across all tumour groups in this study, the time from diagnosis to earliest consultation was 56 days (ranging from 20 days for lung cancer to 82 days for breast cancer). The 2023 provincial BC target of 28 days from referral to earliest consultation was met by all tumour groups, ranging from 18 to 25 days [54]. For radiotherapy specifically, the median time from date of consultation to start radiotherapy across all tumour groups was 40 days. This was prolonged primarily in the prostate cancer group (116 days), and for early- compared to late-stage disease, which is consistent with previously published Canadian data [55]. Compared to retrospective Canadian studies, we found either similar or more prolonged timepoints from diagnosis to first intervention across all tumour groups [60,64,65,67].

There are no studies examining the timepoints to cancer care for Yukon residents compared to BC residents or other Canadians to date. There were two studies which described timepoints to cancer care among British Columbians: Moir et al. (2022), which included all cancer types, breast, prostate and lung cancer, and Van de Vosse et al. (2015), which included lung cancer cases only [57,64]. Compared to BC timepoints in the Moir study, the Yukon cancer cases in this study had longer median timepoints in radiotherapy start time in prostate cancer, but shorter or similar radiotherapy start time in lung cancer and all cancers (no data for breast or colorectal) [57]. There was also shorter or similar chemotherapy start times in all cancers, in lung cancer and in breast cancer (no data for prostate or colorectal) [57]. Perhaps the quicker access to chemotherapy is facilitated by the availability of chemotherapy treatment at Whitehorse General Hospital, under supervision of GPOs [4]. The Van de Vosse study included six lung-specific median timepoints, of which 4/6 of the median timepoints in this study of Yukon cases were more prolonged in duration [64]. Taken together, there is limited and mixed data to compare treatment timepoints of British Columbians and Yukoners, and this is an area that requires further study.

### 4.4. Limitations

This study has several limitations. First, we were unable to capture mortality data, which is important to help understand the impact of access to care and cancer treatments. Mortality data are currently not shared between the Yukon and BC and this will be the focus of future work. Similarly, this study only had access to Yukon health records, which were faxed and added to the BC Cancer medical record, and accordingly, the patient’s comprehensive medical record was not available for review. In addition, approximately 22% of the Yukon’s population self-identify as Indigenous; however, no ethnicity-based data were available in the patient charts [15]. Partnering with First Nations governments and communities to develop more robust and meaningful reporting of cancer data was highlighted as a recommendation from the Government of Yukon, which is an encouraging step forward to help understand cancer care patterns and experiences of this population [2]. Moreover, the study focused exclusively on patients with breast, prostate, colorectal and lung cancer, and we could not account for patients who opted for treatment in other provinces outside of BC or patients who were referred to a cancer centre in BC other than BC Cancer. Location of surgery and systemic therapy was not documented consistently and consequently not collected in this study. Accordingly, it is unclear whether the cancer care timepoints for cases treated exclusively in BC are similar to those for cases treated in both BC and Yukon, and this would be an interesting future study. Thus, although the present study does not provide a comprehensive depiction of the entire Yukon cancer patient population, we focused on the four most common types of cancer as an initial point of study. Finally, while basic treatment data were obtained in this study and helped to serve as a baseline, comprehensive treatment utilization analyses were beyond the scope of this current study. Further work will be underway to better understand cancer treatment utilization patterns amongst this population, compared to BC residents, as a further measure of access to treatment. Future thorough assessments of cross-provincial/territorial data quality and utility can also be conducted to strengthen data-driven policy making and health services delivery.

## 5. Conclusions

In conclusion, this study provides valuable insights into the characteristics and care pathways of cancer patients in the Yukon. There is scarce published data on cancer care amongst Yukon residents and few cancer treatment timepoint benchmarks in Canada. We hope this study will inspire future work to support Yukon cancer care pathways.

## Figures and Tables

**Table 1:** Demographics, tumour characteristics and treatment details of Yukon cancer cases.

	Breast n = 266		Prostate n = 118		Colorectal n = 204		Lung n = 183	
**Sex**								
Male	0	0.0%	118	100.0%	119	58.3%	99	54.1%
Female	266	100.0%	0	0.0%	85	41.7%	84	45.9%
**Yukon Residence**								
Whitehorse	189	71.1%	79	66.9%	137	67.2%	119	65.0%
Other	77	28.9%	39	33.1%	67	32.8%	64	35.0%
**Age**								
Mean	59		67		64		68	
Median	59		67		64		68	
Min	27		51		26		47	
Max	95		84		98		89	
**BC Cancer Referral Site**								
Vancouver	234	88.0%	106	89.8%	>198	>97%	169	92.3%
Other	32	12.0%	12	10.2%	<5	<3%	14	8.7%
**Year of Diagnosis**								
2009–2010	40	15.0%	25	21.2%	20	9.8%	35	19.1%
2011–2012	36	13.5%	20	16.9%	23	11.3%	21	11.5%
2013–2014	35	13.2%	23	19.5%	26	12.7%	27	14.8%
2015–2016	56	21.1%	8	6.8%	33	16.2%	33	18.0%
2017–2019	63	23.7%	29	24.6%	64	31.4%	43	23.5%
2020–2021	36	13.5%	13	11.0%	38	18.6%	24	13.1%
**Stage**								
Early	247	92.9%	97	82.2%	147	72.1%	84	45.9%
Late	19	7.1%	21	17.8%	57	27.9%	99	54.1%
**Histologic Subtype**								
Adeno			118	100.0%	204	100.0%		
IDC	238	89.5%						
ILC	28	10.5%						
Non-squam							106	57.9%
Squamous							42	23.0%
SCLC							35	19.1%
**Grade**								
I	54	20.3%			18	8.8%	<5	<3%
II	>125	>47%			109	53.4%	8	4.4%
III	81	30.5%			18	8.8%	15	8.2%
unknown	<5	<2%	5	4.2%	59	28.9%	>155	>85%
Low (≤6)			5	4.2%				
(3 + 4)			38	32.2%				
(4 + 3)			19	16.1%				
High (≥8)			51	43.2%				
**Surgery**								
Yes	253	95.1%	41	34.7%	149	73.0%	27	14.8%
No	13	4.9%	77	65.3%	55	27.0%	156	85.2%
**Surgery Type (Colorectal only)**								
HC					60	40.3%		
LAR					20	13.4%		
TME					18	12.1%		
APR					11	7.4%		
Other					40	26.8%		
**Chemotherapy**								
Yes	125	47.0%	<5	<4%	127	62.3%	80	43.7%
No	141	53.0%	>113	>96%	77	37.7%	103	56.3%
**Chemotherapy Total Courses**								
	**133**		**<5**		**159**		**83**	
**Chemotherapy Course Intent**								
Curative	100	75.2%	0	0.0%	93	58.5%	27	32.5%
Palliative	33	24.8%	<5	100.0%	66	41.5%	56	67.5%
**Chemotherapy Number of Lines of Palliative Courses**								
Mean	1.2		0.1		1.2		0.6	0.3%
Median	1		1		1		1	0.5%
Max	4		1		5		4	2.2%
**Hormone Therapy**								
Yes	204	76.7%	74	62.7%				
No	17	6.4%	44	37.3%				
N/A	42	15.8%	0	0.0%	204	100.0%	183	100.0%
unknown	3	1.1%	0	0.0%				
**Targeted Therapy**								
Yes	37	13.9%	0	0.0%	43	21.1%	9	4.9%
No	229	86.1%	118	100.0%	161	78.9%	174	95.1%
**Immunotherapy**								
Yes	0	0.0%	0	0.0%	<5	<3%	18	9.8%
No	266	100.0%	118	100.0%	>199	>97%	165	90.2%
**Radiotherapy**								
Yes	153	57.5%	87	73.7%	61	29.9%	125	68.3%
No	113	42.5%	31	26.3%	143	70.1%	58	31.7%
**Radiotherapy Total Completed Courses**								
	**296**		**137**		**95**		**202**	
**Radiotherapy Course Intent**								
Curative	258	87.2%	97	70.8%	66	69.5%	40	19.8%
Palliative	38	12.8%	40	29.2%	29	30.5%	162	80.2%
**Radiotherapy Course Treatment Site**								
Local/regional	262	88.5%	105	76.6%	63	66.3%	102	50.5%
Distant	34	11.5%	32	23.4%	32	33.7%	100	49.5%

Adeno = adenocarcinoma, Non-squam = non-squamous non-small cell lung cancer, Squamous = squamous non-small cell lung cancer, SCLC = small cell lung cancer, IDC = invasive ductal carcinoma, ILC = invasive lobular carcinoma, HC = hemicolectomy, LAR = low anterior resection, TME = total mesorectal excision, APR = abdominoperineal resection.

**Table 2:** Non-referred cases by cancer type, sex, residence, age and stage.

	All		Breast		Prostate		Colorectal		Lung	
Non-referred cases	323		38		150		73		62	
**Sex**										
Male	223	69.0%	0	0%	150	100%	44	60.3%	29	46.8%
Female	100	31.0%	38	100%	0	0%	29	39.7%	33	53.2%
**Yukon Residence**										
Whitehorse	216	66.9%	23	60.5%	99	66.0%	48	65.8%	46	74.2%
Other	90	27.9%	7	18.4%	>45	>30.0%	>20	>27%	>10	>16%
Unknown	17	5.3%	8	21.1%	<5	<3%	<5	<7%	<5	<8%
**Age**										
Mean	68		66		67		68		69	
Median	68		64		67		68		71	
Minimum	32		37		48		32		40	
Maximum	91		91		87		87		86	
**Not referred with known stage at diagnosis**	250		25		120		57		48	
Early-stage	153	61.2%	20	80.0%	68	56.7%	40	70.2%	25	52.1%
Late-stage	97	38.8%	5	20.0%	52	43.3%	17	29.8%	23	47.9%

## Data Availability

The dataset used and analyzed during the current study were obtained from the British Columbia and Yukon Cancer Registries and are not publicly available due to privacy legislation and institutional data sharing agreements. Data, however, can be requested through a data access request to BC Cancer following their processes at http://www.bccancer.bc.ca/health-professionals/professional-resources/bc-cancer-registry/request-registry-data (accessed on 5 December 2023). Requests to access these datasets should be directed to datareq@bccancer.bc.ca.

## Supplementary Materials

The following supporting information can be downloaded at https://www.mdpi.com/article/10.3390/curroncol32110641/s1, Table S1: Mutation Status of Breast, Colorectal and Lungs Cases; Table S2a–e. Canadian published median target timepoints, median timepoints from retrospective studies and median timepoints from our study by tumour group and cancer stage at diagnosis.

## Abbreviations

The following abbreviations are used in this manuscript:

BC	British Columbia
NSCLC	Non-small cell lung cancer
HER2	Human epidermal growth factor receptor 2
KRAS	Kirsten rat sarcoma virus
MMR	Mismatch repair
EGFR	Epidermal growth factor receptor
PDL1	Programmed death ligand 1
PSA	Prostate specific antigen
GPO	General Practitioners in Oncology
