# Supplementary material for: Cancer Treatment Patterns Among Yukon Residents Referred to British Columbia for Care: A 13-Year Retrospective Study
Source: Curr Oncol. 2025 Nov 16;32(11):641. doi: 10.3390/curroncol32110641 (PMC12651239; doi:10.3390/curroncol32110641)
Supplement: Supplementary file 1 [file curroncol-32-00641-s001.zip › curroncol-3943050-supplementary.pdf]

Current Oncology

Supplemental tables

Table S1. Mutation Status of Breast, Colorectal and Lungs Cases

Breast n=266	
**Tumour Marker**	**n**
HR+ HER2+	24 (9.0%)
HR+HER2-	199 (74.3%)
HR-HER2+	>10 (>4%)
HR-HER2-	28 (10.4%)
Unknown	<5 (<2%)

Colorectal n=204		
**Tumour Marker**	**Status**	**n**
KRAS	Positive	20 (9.8%)
	Negative	39 (19.1%)
	Unknown	145 (71.1%)
BRAF	Positive	6 (2.9%)
	Negative	44 (21.6%)
	Unknown	154 (75.5%)
MMR	Positive	23 (11.3%)
	Negative	63 (30.9%)
	Unknown	118 (57.8%)

Lung n=183, of which NSCLC=148 and non-squamous n=106		
**Tumour Marker**	**Status**	**n**
EGFR	Positive	<5 (<5%)
	Negative	>45 (>42%)
	Unknown	55 (51.9%)
KRAS	Positive	16 (15.1%)
	Negative	13 (12.3%)
	Unknown	77 (72.6%)
G12C	Positive	7 (6.6%)
	Negative	22 (20.8%)
	Unknown	77 (72.6%)
ALK	Positive	0 (0.0%)
	Negative	46 (43.4%)
	Unknown	60 (56.6%)
ROS1	Positive	0 (0.0%)
	Negative	31 (29.2%)
	Unknown	75 (70.8%)
PDL1	<1%	13 (12.3%)
	1-49%	7 (6.6%)
	>50%	16 (15.1%)
	Unknown	70 (66.0%)
PDL1 (all NSCLC)	<1%	13 (8.8%)
	1-49%	12 (8.1%)
	>50%	21 (14.2%)
	Unknown	102 (68.9%)

Table 2a-e. Canadian published median target timepoints, median timepoints from retrospective studies and median timepoints from our study by tumour group and cancer stage at diagnosis. The asterisk* indicates BC specific data

			**All Cancers Published Median Timepoints, And Median Timepoints Of This Study's Four Cancer Groups**					
Timepoints	**Target**	**Published data**	All		Early Stage		Late Stage	
			n	Median	n	Median	n	Median
First Investigation to Bx			440	30	328	31	112	26
Bx to Surgery			418	43	395	44	22	29
Bx to Rx			647	35	493	44	152	12
Bx to First Intervention			639	45	495	47	142	32
Bx to Earliest Consult			702	56	515	68	185	29
Rx to Earliest Consult	14[,1],28[2*]		743	21	551	23	190	19
Rx to RO Consult			524	29	393	32	130	21
Rx to MO Consult			590	22	418	24	170	21
RO Consult to RT Start	10,14[1,3]	28[4*]	420	40	309	52	110	7
MO Consult to CT Start		33[4*]	195	16	143	16	51	14

			**Breast Cancer Median Timepoints**					
Timepoints	**Target**	**Published data**	All		Early Stage		Late Stage	
			n	Median	n	Median	n	Median
First Investigation to Bx	42[5,6]	28[7],39[8]	233	23	217	23	16	22
Bx to Surgery		17[9]	248	42	238	42	10	32
Bx to Rx			247	56	232	59	15	40
Bx to First Intervention		35[7]	255	41	239	42	16	32
Bx to Earliest Consult			260	82	242	83	18	47
Rx to Earliest Consult			252	22	235	22	17	20
Rx to RO Consult			182	44	171	47	11	21
Rx to MO Consult	14[5,6]		228	21	213	22	15	20
RO Consult to RT Start	28[5]	7-35[4]	153	50	144	52	9	20
MO Consult to CT Start	28[5]	1-35[4],35[4*]	63	17	57	18	6	7

			**Prostate Cancer Median Timepoints**					
Timepoints	**Target**	**Published data**	All		Early Stage		Late Stage	
			n	Median	n	Median	n	Median
First Investigation to Bx			55	91	42	93	13	63
Bx to Surgery		54[10]	33	98	33	98	0	-
Bx to Rx			105	36	90	37	15	32
Bx to First Intervention		73[7]	99	97	83	98	16	29
Bx to Earliest Consult			111	61	92	64	19	52
Rx to Earliest Consult			113	25	95	28	18	17
Rx to RO Consult			109	28	94	29	15	15
Rx to MO Consult			21	25	10	54	11	20
RO Consult to RT Start		28[4]*, 7-70[4]	84	116	70	117	14	78
MO Consult to CT Start			<5	0	0	-	<5	0

			**Colorectal Cancer Median Timepoints**					
Timepoints	**Target**	**Published data**	All		Early Stage		Late Stage	
			n	Median	n	Median	n	Median
First Investigation to Bx			0	-	0	-	0	-
Bx to Surgery		28[10]	124	38	113	41	10	21
Bx to Rx			175	25	121	33	52	14
Bx to First Intervention		37[7]	155	41	117	38	36	47
Bx to Earliest Consult			177	50	120	55	55	35
Rx to Earliest Consult			200	24	138	25	60	22
Rx to RO Consult			79	26	55	26	23	31
Rx to MO Consult			193	25	131	26	60	22
RO Consult to RT Start			60	14	40	18	19	7
MO Consult to CT Start			75	14	58	15	16	14

Lung Cancer Median Timepoints								
Timepoints	Target	Published data	All		Early Stage		Late Stage	
			n	Median	n	Median	n	Median
First Investigation to Bx		27[11*], 38[12] 42[13] ,49[14]	152	35	69	45	83	25
Bx to Surgery		16[10]18[11*], 45[14]	13	49	11	49	<5	69
Bx to Rx		8*[11]	120	9	50	13	70	13
Bx to First Intervention		31[14],35[12], 26[11*], 43[7]	130	37	56	48	74	29
Bx to Earliest Consult		18*[11]	154	20	61	23	93	19
Rx to Earliest Consult		10[11*]	178	18	83	20	95	15
Rx to RO Consult			154	21	73	23	81	18
Rx to MO Consult			148	21	64	22	84	21
RO Consult to RT Start		7-28[4], 28[4*]	123	15	55	52	68	7
MO Consult to CT Start		10-31[4], 31[4*]	56	19	28	17	28	24

RO = radiation oncology, MO = medical oncology, RT =radiotherapy, CT=chemotherapy, Bx=biopsy, Rx =referral

References

(1) Wait Time Alliance. Unfinished Business Report Card on Wait Times in Canada, 2009. https://caro-acro.ca/wp-content/uploads/2016/10/Wait-Time-Alliance-Report-Card-1.pdf (accessed 2024-11-23).

(2) Harnett, C. *B.C. premier says faster progress needed on cancer care delivery*. Times Colonist. https://www.timescolonist.com/local-news/premier-says-faster-progress-needed-on-cancer-care-delivery-8042767 (accessed 2024-11-23).

(3) Wong, F. *Manpower-and-Standards-of-Care-in-Radiation-Oncology-Committee-Definition-of-RT-Waiting-September-2000*. Canadian Association of Radiation Oncologists. https://caro-acro.ca/wp-content/uploads/2016/10/Manpower-and-Standards-of-Care-in-Radiation-Oncology-Committee-Definition-of-RT-Waiting-September-2000.pdf (accessed 2024-11-23).

(4) Moir, M.; Barua, B. *Waiting Your Turn: Wait Times for Health Care in Canada, 2022 Report*. Fraser Institute. https://bit.ly/3I1dgRx (accessed 2024-11-23).

(5) Canadian Partnership Against Cancer. Pan-Canadian Standards: Breast Cancer Surgery, 2019. https://s22457.pcdn.co/wp-content/uploads/2019/04/Breast-Cancer-Surgery-Standards-Reports-EN-2019.pdf (accessed 2024-11-23).

(6) Prashad, A.; Mitchell, M.; Argent-Katwala, M.; Daly, C.; Earle, C. C.; Finley, C. Pan-Canadian Standards for Cancer Surgery. *Can J Surg* **2019**, *62* (4 Suppl 3), S171–S183. https://doi.org/10.1503/cjs.010419.

(7) Chaudhry, M.; Kandasamy, S.; Habbous, S.; Chan, C.; Barisic, A.; Faisa, A.; Walker, M.; Haque, M.; Sheppard, A.; Uri, E.; Ashu, E.; Schwartz, N.; Young, S.; McCurdy, B. Cancer System Quality Index 2021: Ontario Cancer System Performance, 2021. https://www.ontariohealth.ca/sites/ontariohealth/files/csqi-report-2021.pdf.

(8) Chiarelli, A. M.; Muradali, D.; Blackmore, K. M.; Smith, C. R.; Mirea, L.; Majpruz, V.; O'Malley, F. P.; Quan, M. L.; Holloway, C. M. Evaluating Wait Times from Screening to Breast Cancer Diagnosis among Women Undergoing Organised Assessment vs Usual Care. *Br J Cancer* **2017**, *116* (10), 1254–1263. https://doi.org/10.1038/bjc.2017.87.

(9) Plotogea, A.; Chiarelli, A. M.; Mirea, L.; Prummel, M. V.; Chong, N.; Shumak, R. S.; O'Malley, F. P.; Holloway, C. M. B. Factors Associated with Wait Times across the Breast Cancer Treatment Pathway in Ontario. *Springerplus* **2013**, *2*, 388. https://doi.org/10.1186/2193-1801-2-388.

(10) Grunfeld, E.; Watters, J. M.; Urquhart, R.; O'Rourke, K.; Jaffey, J.; Maziak, D. E.; Morash, C.; Patel, D.; Evans, W. K. A Prospective Study of Peri-Diagnostic and Surgical Wait Times for Patients with Presumptive Colorectal, Lung, or Prostate Cancer. *Br J Cancer* **2009**, *100* (1), 56–62. https://doi.org/10.1038/sj.bjc.6604819.

(11) Van de Vosse, D.; Chowdhury, R.; Boyce, A.; Halperin, R. Wait Times Experienced by Lung Cancer Patients in the BC Southern Interior to Obtain Oncologic Care: Exploration of the Intervals from First Abnormal Imaging to Oncologic Treatment. *Cureus* **2015**, *7* (9), e330. https://doi.org/10.7759/cureus.330.

(12) Common, J. L.; Mariathas, H. H.; Parsons, K.; Greenland, J. D.; Harris, S.; Bhatia, R.; Byrne, S. C. Reducing Wait Time for Lung Cancer Diagnosis and Treatment: Impact of a Multidisciplinary, Centralized Referral Program. *Canadian Association of Radiologists Journal* **2018**, *69* (3), 322–327. https://doi.org/10.1016/j.carj.2018.02.001.

(13) Byrne, S. C.; Barrett, B.; Bhatia, R. The Impact of Diagnostic Imaging Wait Times on the Prognosis of Lung Cancer. *Canadian Association of Radiologists Journal* **2015**, *66* (1), 53–57. https://doi.org/10.1016/j.carj.2014.01.003.

(14) Denault, M.-H.; Labbé, C.; St-Pierre, C.; Fournier, B.; Gagné, A.; Morillon, C.; Joubert, P.; Simard, S.; Martel, S. Wait Times and Survival in Lung Cancer Patients across the Province of Quebec, Canada. *Curr Oncol* **2022**, *29* (5), 3187–3199. https://doi.org/10.3390/curroncol29050259.
